# Mortality in polymyalgia rheumatica: a 38-year prospective population-based cohort study from Southern Norway

**DOI:** 10.1186/s13075-025-03613-9

**Published:** 2025-07-21

**Authors:** Stig Tengesdal, Øyvind Molberg, Øyvind Holme, Jan Tore Gran, Geirmund Myklebust

**Affiliations:** 1 Department of Research, Sorlandet Hospital, Egsveien 100, Kristiansand, 4615 Norway; 2 Institute of Clinical Medicine, University of Oslo, Oslo, Norway; 3 Department of Rheumatology, Oslo University Hospital, Rikshospitalet, Oslo Norway; 4 Institute of Health and Society, University of Oslo, Oslo, Norway

**Keywords:** Polymyalgia rheumatica, Giant cell arteritis, Epidemiology, Mortality, Survival

## Abstract

**Background:**

Robust long-term mortality data on patients with polymyalgia rheumatica (PMR) are lacking. The aim of this study was to determine all-cause mortality in isolated PMR using a large, population-based, inception cohort followed prospectively over a 38-year period.

**Methods:**

Between 1987 and 1997, 337 incident cases of PMR and biopsy-proven GCA were included in a prospective, population-based inception cohort in Aust-Agder County, Norway. Diagnosis was ascertained clinically by a rheumatologist, with PMR cases meeting Bird`s criteria. Patients were followed until death or end of study on December 31st, 2024. Each case was matched by gender, age at inclusion, and residency with 15 population comparators drawn from the population registry in Norway. We assessed mortality and survival by standard mortality ratios (SMR) and the Kaplan-Meier method.

**Results:**

A total of 274 patients with isolated PMR (66.1% female, mean age at diagnosis 71.9 years) and 63 patients with GCA (76.2% female, mean age at diagnosis 71.6 years) were included. By the end of the study, 96.4% of all patients were deceased. Mean follow-up time for all patients was 13.7 years, with a maximum of 35.3 years. For cases with isolated PMR, the overall SMR was 0.97 (95% confidence interval [CI] 0.85, 1.09), for men 0.77 (95% CI 0.62, 0.95), and for women 1.11 (95% CI 0.95, 1.28). For GCA, the overall SMR was 1.10 (95% CI 0.85, 1.40), with no gender difference.

**Conclusions:**

In this comprehensive long-term follow-up study with nearly complete data on mortality, isolated PMR was not associated with increased mortality, reinforcing the view that it does not confer a higher mortality risk.

## Introduction

Polymyalgia rheumatica (PMR) is a common rheumatic disease in the elderly. It is characterized by pain and stiffness in the shoulders and pelvic girdles, accompanied by systemic inflammation [1]. Onset of PMR is typically between the ages of 70 and 80 years, with women representing around 75% of those affected [2]. Disease onset before the age of 50 is exceptionally rare. Individuals of Scandinavian and other Northern European ancestry have higher predisposition to PMR compared to those of other ethnic backgrounds. Accordingly, the highest reported incidence rates for PMR are from Northern Europe and range from 34 to 113 cases per 100 000 people aged ≥ 50 years [3]. PMR and giant cell arteritis (GCA) are frequently overlapping conditions, and part of the same inflammatory disease spectrum [4]. In a systematic review and meta-analysis, the observed prevalence of concurrent GCA in PMR was 22% [5]. For isolated PMR, the cornerstone of treatment is still glucocorticoids (GC), with an initial medium range daily doses (equivalent to 15–20 mg prednisolone) typically providing rapid relief of symptoms. The optimal dose reduction rate for GC is not clear. Neither is the duration of GC treatment, but available data indicates that most patients require GC-treatment for at least two years [6]. GCs have several known side effects, and GC-related comorbidities are found to be increased in PMR [7].

Despite presence of systemic inflammation and prolonged GC treatment, with associated comorbidities, it appears from previous studies that mortality rate in PMR is comparable to or even lower than that of the general population [8–11]. As most of the studies assessing mortality in PMR have been retrospective and included non-incident cases, the ability to draw definitive conclusions is limited.

To our knowledge, only one population-based, prospective study on mortality in PMR has been conducted to date [8]. This study was part of a larger clinical and epidemiological PMR project in Norway conducted up to 2003 [12–15]. However, a major limitation of this study was its relatively short follow-up period and the low number of deaths. Hence, there is a distinct lack of prospective longitudinal studies examining the long-term outcome and mortality rate in PMR.

The aim of the present study was to determine the long-term all-cause mortality in the population-based incident PMR cohort from Norway referred to above [8]. Mortality rates in the PMR cohort were compared with individually matched population comparators, as well as with an incident GCA cohort diagnosed and followed during the same period.

## **Methods**

### Study design and patient population

Between 1987 and 1997, a total of 398 cases of PMR and GCA from Aust-Agder County, Southern Norway, were enrolled in a population-based cohort study [8, 12]. At the time, the county had only one rheumatology department and no private practicing rheumatologists. All general practitioners in the county were informed about the study in advance and asked to refer all new cases. The patients were diagnosed clinically by two experienced rheumatologists. PMR cases fulfilled the criteria suggested by Bird et al. [16], and GCA diagnosis was confirmed with a positive temporal artery biopsy (TAB). Further details about the inclusion process has been published previously [8]. Clinical data were regularly collected and monitored up until 1997, during which the cohort was extensively studied. The list of individuals from the patient inception cohort was maintained by the Hospital of Southern Norway, Kristiansand for quality assurance purposes. It included the personal identification numbers, gender, diagnoses (PMR and/or GCA), inclusion dates, and residency information at the time of inclusion.

In the present follow-up study, all cases with isolated PMR and with a biopsy-proven GCA from the inception cohort were identified and followed until death or December 31, 2024. Cases in which PMR and GCA coexisted were categorized as GCA, as these patients required higher initial and maintenance GC-doses and were regarded to be at increased risk of GCA-related complications [17].

### Matched comparator group

In Norway every resident is assigned a unique national registration number at birth, which is linked to their sex and date of birth. This number allows for identification within national registries and hospital databases, ensuring reliable data tracking. For this study, we set up a matched comparator group drawn from Norway’s National Population Registry (NPR). The comparator group included in total 4110 individuals, 15 per PMR and GCA index case. We matched each index case to its 15 comparators by four parameters: (i) year of birth (ii) gender (iii) vital status at time of study entry (alive when their corresponding index case was included in the study), and (iv) residency in same county (Agder) at time of study entry.

### Data assessment

Inclusion dates for cases were validated by cross-referencing patient records from the original study and reviewing medical charts. Additional information regarding disease characteristics and treatment for the patient cohort could not be retrieved, as only a limited amount of data from the inclusion period has been digitized.

We obtained vital status and date of death for both patients and comparators from the NPR. Emigration status and corresponding dates were also acquired from the NPR, with individuals who emigrated being censored at their date of emigration.

### Statistical analysis

Descriptive statistics were used to summarize the sample characteristics. Group differences for continuous variables were assessed using the T-test if parametric or the Mann-Witney-Wilcoxon test if nonparametric data.

Ten-year-specific person-years of follow-up were calculated from the date of diagnosis until death or end of study. Mortality was assessed using standard mortality ratios (SMR), determined by dividing the observed number of deaths to the expected number. The expected number of deaths was calculated by multiplying the cases person-years by the death rates of the matched comparator population. The 95% confidence intervals (CI) for SMRs were calculated using the Mid-P exact test.

Overall cumulative survival for both cases and comparators were predicted using Kaplan-Meier plots. Death was registered as the event (outcome), and time-to-event data were censored either at study end (December 31, 2024) or at the time of emigration for patients or comparators who emigrated. Differences between groups were assessed using the log-rank test. If the Kaplan-Meier plot indicated any early differences between groups, the Gehan-Breslow-Wilcoxon test was additionally performed.

Statistical analyses and graphical plots were conducted using Stata version 18.0 (StataCorp LLC, Texas, USA) and GraphPad version 10 (GraphPad Software, Boston, Massachusetts, USA). P values less than 0.05 were considered statistically significant.

### Ethics

This study was approved by the regional ethics committee with exemption from informed consent for identifications of patients and linkage to the NPR (Case number 45964, November 14, 2019).

## Results

### Patient characteristics and outcomes

**Fig. 1:**
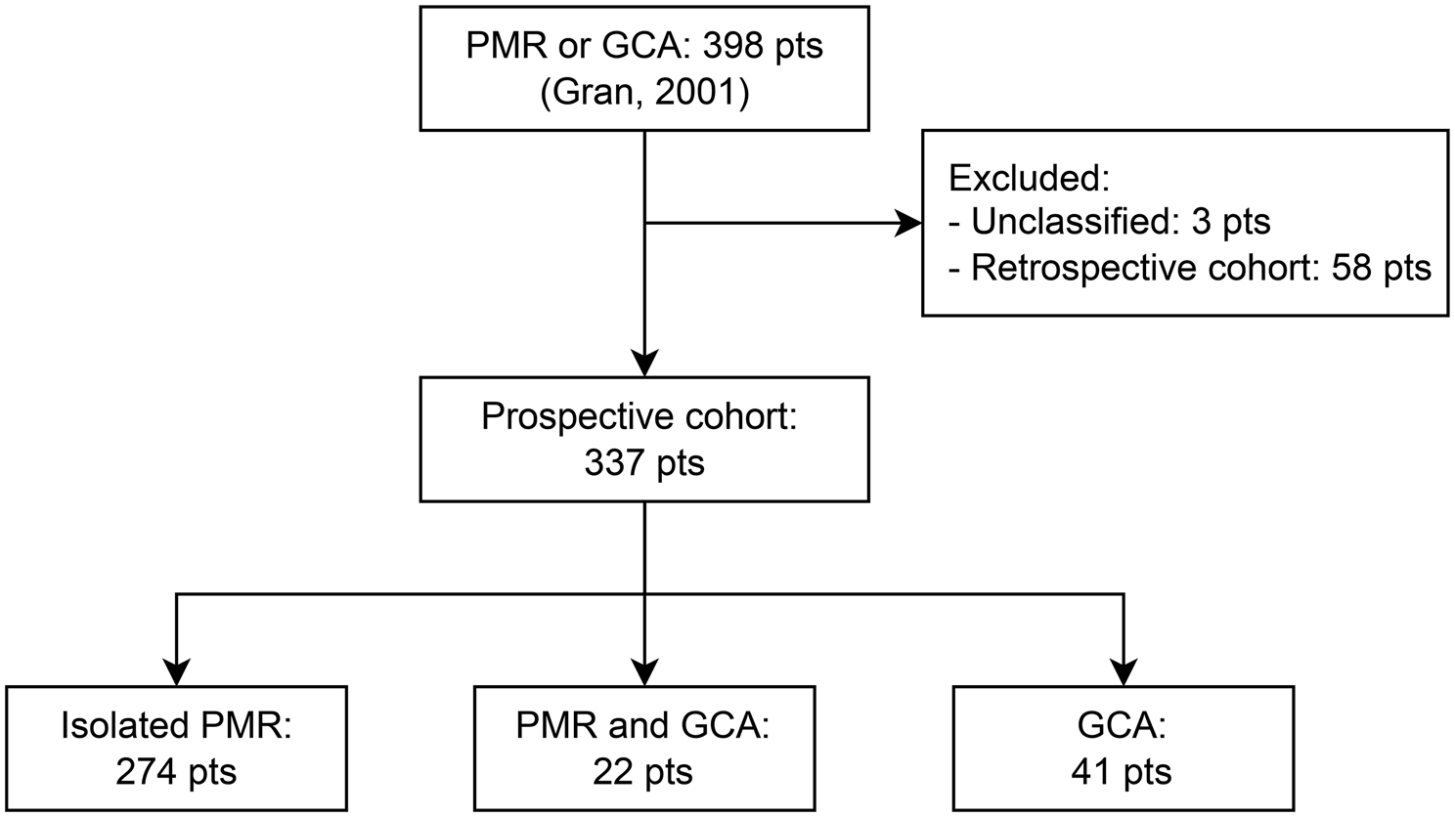
Flowchart illustrating relationships between the original project cohort and incident PMR and GCA cohorts studied

Among the incident cases, we identified 274 cases of isolated PMR and 63 cases of GCA, of which 22 had coexisting PMR (Fig. 1). During the verification of registered inclusion dates, a review of medical records revealed that nine patients (6 with isolated PMR, 3 with GCA with PMR) had been included between 1985 and 1986. These patients were included in the inception cohort, and comparators were matched according to previously defined four parameters. One of the cohort patients and 6 of the matched comparators emigrated and were therefore censored at date of emigration. The baseline characteristics and outcomes parameters for cohort cases and the matched comparator group are summarized in Table 1. 

**Table 1:** Baseline characteristics and outcome parameters of cohort cases and matched comparator group

	Cohort patients			Matched comparator group		
	Total	Men	Women	Total	Men	Women
**Baseline**						
**Isolated PMR**						
- Number of cases/comparators (%)	274	93 (33.9)	181 (66.1)	4110	1395 (33.9)	2715 (66.1)
- Mean age at diagnosis / inclusion (± SD)	71.9 (8.4)	71.2 (8.5)	72.2 (8.3)	71.9 (8.3)	71.2 (8.5)	72.2 (0.3)
**GCA**						
- Number of cases/comparators (%)	63	15 (23.8)	48 (76.2)	945	225 (23.8)	720 (76.2)
- Mean age at diagnosis / inclusion (± SD)	71.6 (6.8)	70.4 (6.9)	72.0 (6.8)	71.6 (6.8)	70.4 (6.6)	72.0 (6.8)
**Outcomes**						
**Isolated PMR**						
- Deceased*, n (%)	263 (96.0)	89 (95.7)	174 (96.1)	3944 (96.0)	1360 (97.5)	2584 (95.2)
- Mean age at death, years (SD)	85.7 (7.3)	85.1 (7.2) †	85.9 (7.3)	85.4 (7.8)	83.1 (7.9) †	86.5 (7.5)
- Mean follow-up time, years (95% CI)	14.0 (13.0,15.0)	14.2 (12.4, 16.0) †	13.9 (12.7, 15.1)	13.7 (13.4, 14.0)	12.0 (11.5, 12.5) †	14.6 (14.2, 14.9)
**GCA**						
- Deceased*, n (%)	61 (96.8)	14 (93.3)	47 (97.9)	921 (97.5)	225 (100%)	696 (96.7)
- Mean age at death, years (SD)	84.4 (7.4)	83.6 (6.3)	84.7 (7.7)	84.7 (7.5)	82.6 (7.4)	85.4 (7.4)
- Mean follow-up time, years (95% CI)	12.5 (10.6, 14.4)	12.0 (7.6, 16.3)	12.7 (10.5, 14.8)	13.3 (12.7, 13 − 8)	12.1 (11.0, 13.2)	13.6 (13.0, 14.2)

* at the censoring date of December 31, 2024.

† Significant difference between cases and comparators (*P* < 0.05).

Abbreviations: PMR, polymyalgia rheumatica; GCA, giant cell arteritis; n, number; SD, standard deviation; CI, confidence interval

**Fig. 2:**
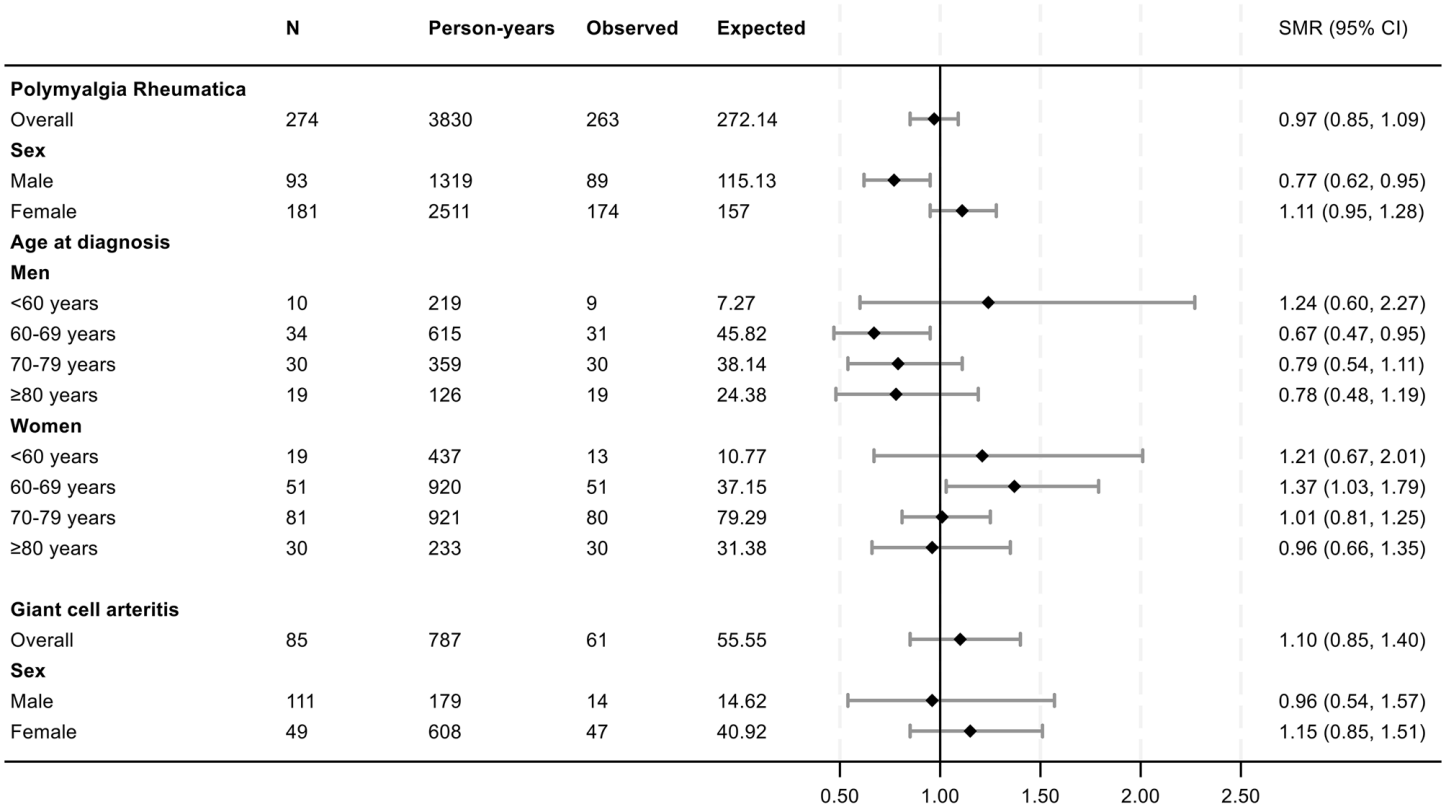
Forest plot of SMRs for new-onset PMR or GCA compared with the matched comparators. Abbreviations: SMR: standard mortality ratios; N: number of patients; Observed: number of deaths in the PMR and GCA patient cohorts; Expected: number of expected deaths estimated from the total number of deaths observed in the age-, gender and residency-matched population comparator group

**Fig. 3:**
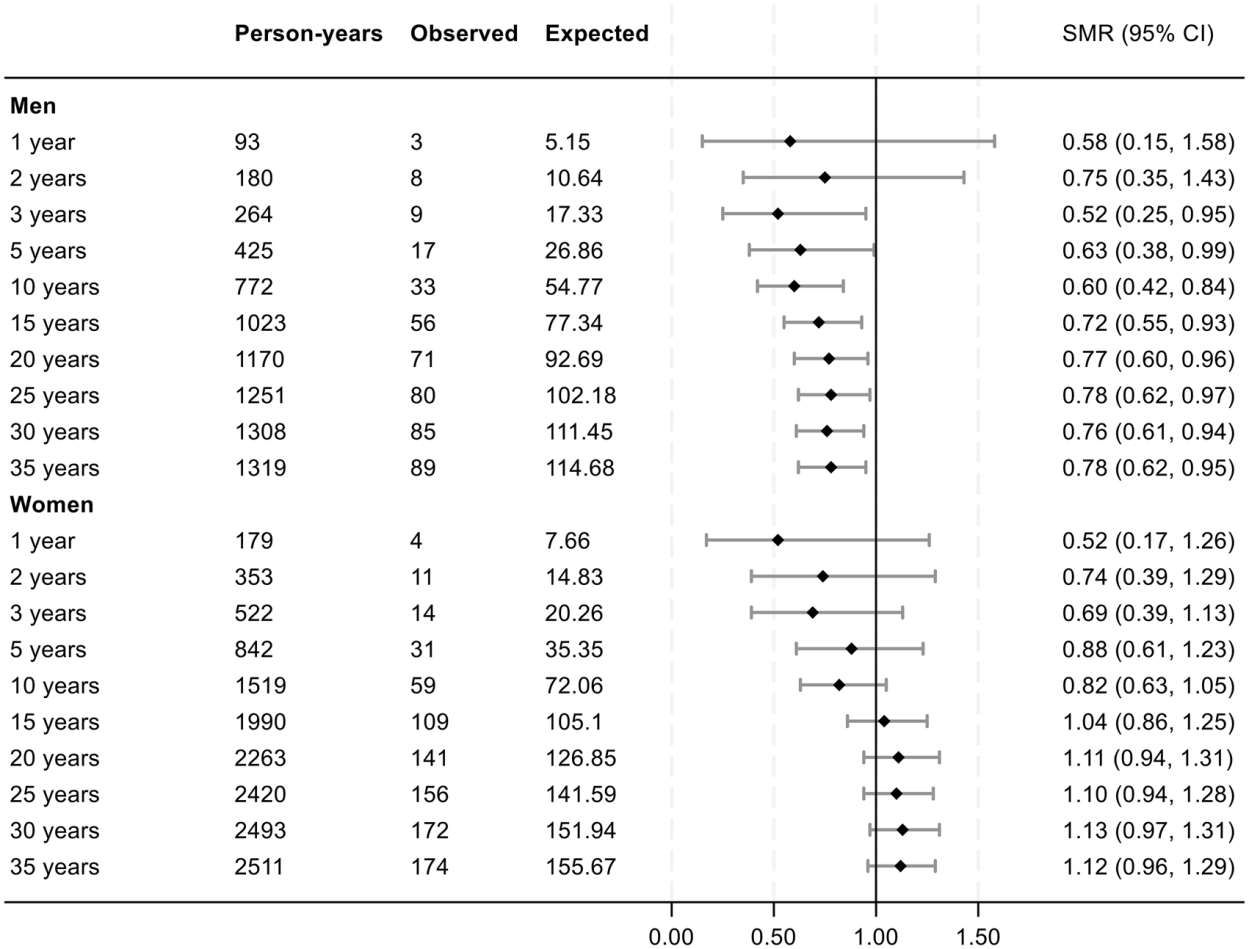
Forest plot of SMRs across the observation period in the prospective PMR cohort. Data are expressed as years of follow-up from diagnosis of PMR. Abbreviations: SMR: standard mortality ratios; N: number of patients; Observed: cumulative number of deaths in the PMR patient cohort at the defined follow-up times. Expected: cumulative number of expected deaths in the in age-, gender and residency-matched population comparator group, estimated from total number of deaths observed in this group at the different lengths of follow-up

**Fig. 4:**
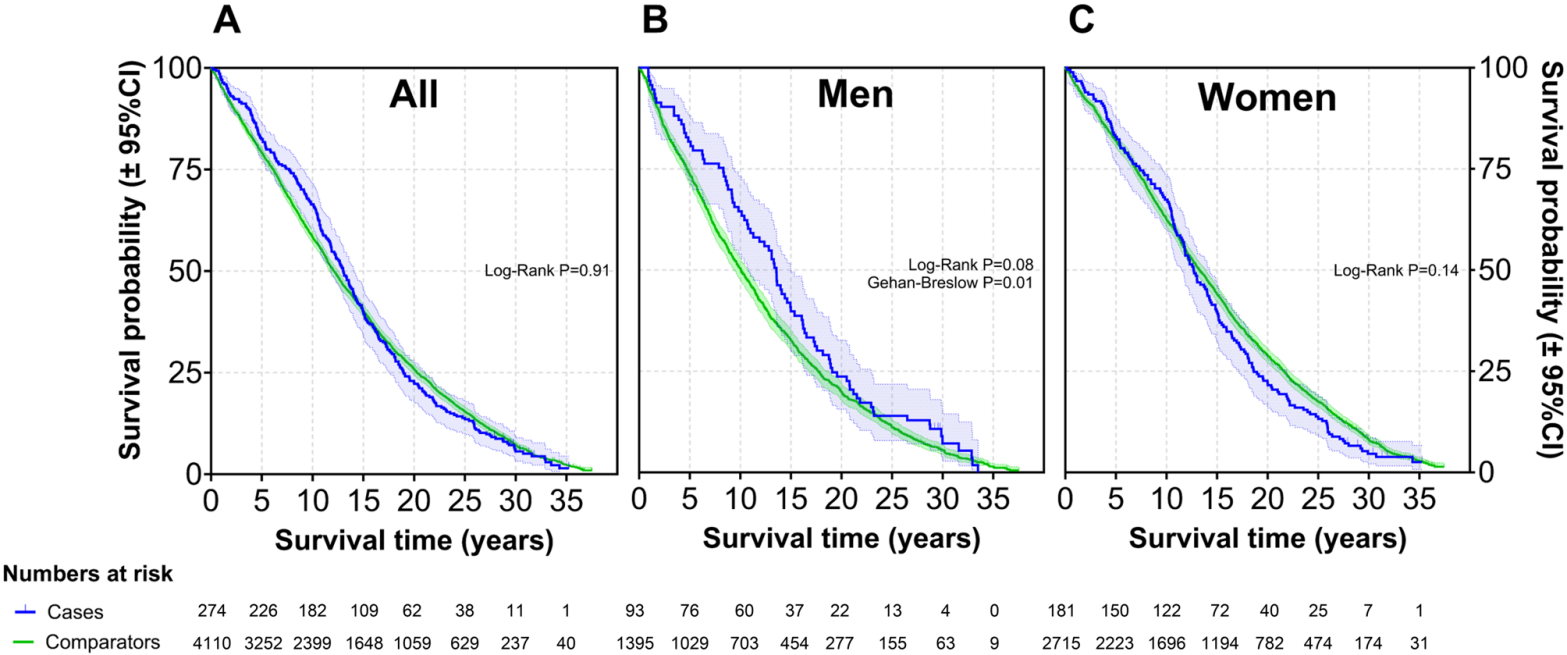
Kaplan-Meier survival curves for patients with new-onset PMR compared to matched comparators. Legend: (**A**) All patients, (**B**) Men, and (**C**) Women

**Fig. 5:**
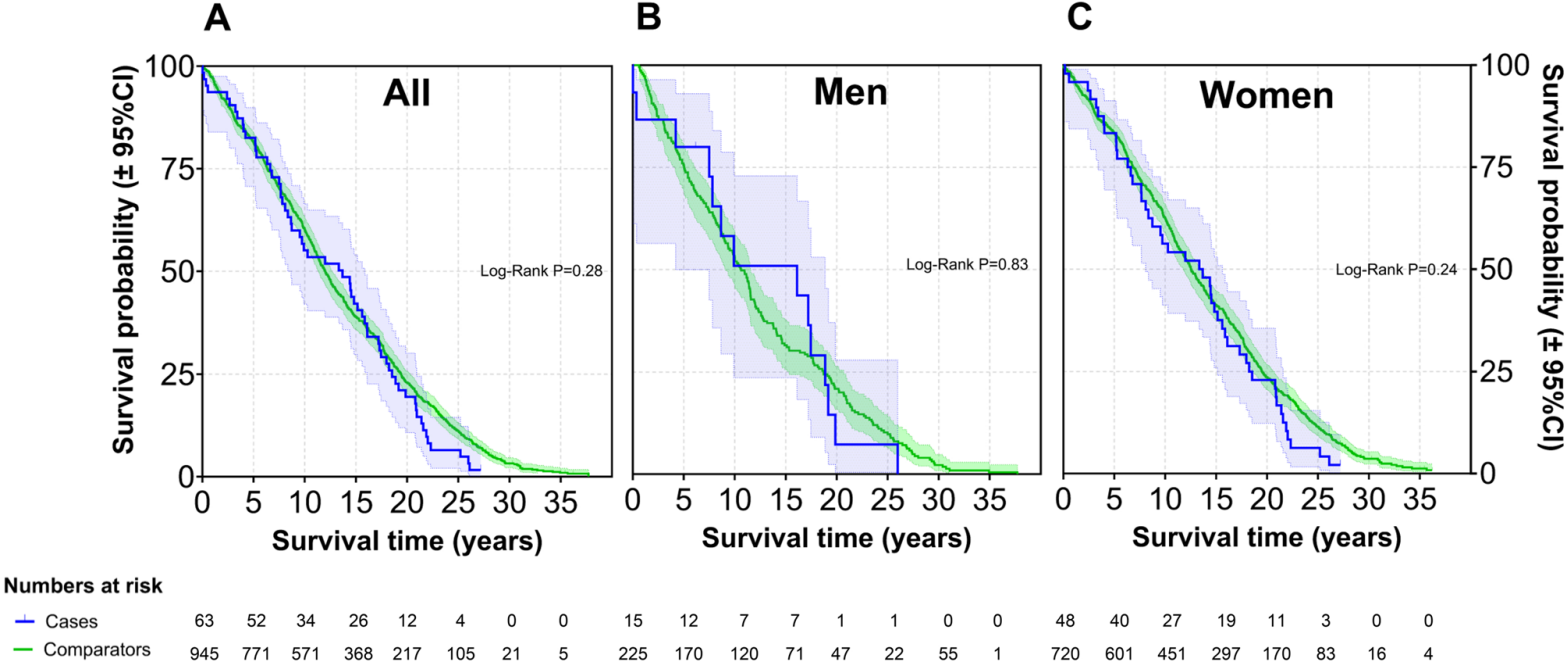
Kaplan-Meier survival curves for patients with new-onset GCA compared to matched comparators. Legend: (**A**) All patients, (**B**) Men, and (**C**) Women

Among the 274 cases with isolated PMR, 181 (66.1%) were female. The mean age at diagnosis was 71.9 years (range 52.0 to 90.6). Mean follow-up time from diagnosis to the end of study for all PMR patients was 14.0 (range 0.2 to 35.3) years. By the end of the study period, 263 (96%) of the individuals in the PMR cohort had died. The mortality risk in patients with PMR and GCA, expressed as SMR, is presented in Figs. 2 and 3. For patients with isolated PMR, the overall SMR was 0.97 (95% CI 0.85, 1.09). Among females, the SMR was 1.11 (95% CI 0.95, 1.28), while it was 0.77 (95% CI 0.62, 0.95) in men. For patients with isolated PMR, overall cumulative survival did not differ significantly from the matched comparators (Log-Rank *P* = 0.91, Fig. 4). In the GCA cohort (*n* = 63), the overall SMRs was 1.10 (95% CI 0.85, 1.40), for men 0.96 (95% CI 0.54, 1.57) and women 1.15 (95% CI 0.85, 1.51). Cumulative survival did not differ from comparators (Log-Rank *P* = 0.28, Fig. 5).

## Discussion

To our knowledge, the present study is the first prospective, population-based cohort study with long-term follow-up and near complete mortality data for PMR. We observed that all-cause mortality was not increased among the patients with isolated PMR or biopsy-proven GCA compared to matched population comparators over the 38-year study period. On the contrary, men with isolated PMR demonstrated significantly lower mortality rates.

There has been a paucity of robust data on the long-term outcomes of PMR. To contextualize the findings of our study, we have compiled an overview of key data from previous major studies evaluating mortality and survival in PMR cohorts (Table 2). As shown in the table, several epidemiologic studies from Olmsted County, Minnesota, USA have reported no increased mortality in PMR patients over extended follow-up periods [9, 10, 18]. Similarly, a more recent study from the UK by Partington et al., which included a large sample of PMR patients found no significant impact on life expectancy between PMR patients and matched controls [11]. In contrast, a 1995 study from Gothenburg, Sweden, reported increased mortality among 220 PMR patients [19]. Limitations to the prior studies includes potential for misclassification bias [9–11, 18], short follow-up time and a low proportion of deaths occurring during the study period [8, 19].

**Table 2:** Characteristics of previous studies and current study on mortality and survival in polymyalgia rheumatica

Author and publication year	Location and study period	Study design	Source	Case validation	*N* (n of deaths)	Controls	Mean observation time	Outcome
Salvarani, 1995 (18)	Olmsted County, Minnesota, USA 1970–1991	Retrospective cohort study	Case record linkage, population based	Clinical diagnosis	245 (NR)	Expected rates	NR	Lower mortality for men, *P* = 0.003. No difference for women.
Schaufelberger, 1995 (19)	Gothenburg, Sweden 1985–1989	Retrospective cohort study	Pathology register and case record linkage	Clinical diagnosis and negative TAB for GCA	220 (41)	Matched rates	36 months	Increased mortality: MRR 1.43, *P* < 0.005), especially for men with vascular diseases the first 2 years of the disease.
Gran, 2001 (8)	Aust-Agder County, Southern Norway 1987–1997	Retro- and prospective cohort study	Population-based, incident cases	Clinical diagnosis and Bird’s criteria, 1979	315 Prospective: 274 (56) Retrospective: 41 (NR)	Matched rates	64 months	Lower mortality: RR 0.73, *P* = 0.03.
								RR prospective pts: 0.70 (95% CI 0.52, 0.95).
Doran, 2002 (9)	Olmsted County, Minnesota, USA 1970–1999	Retrospective cohort study	Case record linkage, population based	Clinical diagnosis	378 (NR)	Expected rates	8.7 years	No difference: SMR 0.88 (95% CI 0.75, 1.01), Log-Rank *P* = 0.06.
Raheel, 2017 (10)	Olmsted County, Minnesota, USA 2000–2014	Retrospective cohort study	Case record linkage, population based	2012 ACR/EULAR classification criteria	377 (107)	Expected rates	6.6 years	Lower mortality: SMR 0.70 (95% CI 0.57, 0.85).
Partington, 2020 (11)	United Kingdom 1990–2016	Retrospective cohort study	National primary care database	Diagnostic codes, GC prescriptions	18,943 (6,046)	Matched rates	8.0 years	No difference: MRR 1.00 (95% CI 0.97, 1.03).
Huo, 2024 (35)	USA 1999-2020	Retrospective cohort study	Centers for Disease Control and Prevention	Death certificate with diagnostic codes (ICD-10)	21,136 (21,136)	Expected rates	NR	PMR listed as underlying cause of death: ASMR (per 100,000) for F:M: 1.8-5.1:1. PMR not underlying cause of death: ASMR (per 100,000) for F:M: 1.8-3.3:1.
Tengesdal, 2025	Aust-Agder County, Southern Norway 1987-2024	Prospective cohort study	Population-based, incident cases	Clinical diagnosis and Bird’s criteria, 1979	274 (263)	Matched rates	14.0 years	Lower mortality for men: SMR 0.77 (95% CI 0.62, 0.95). No difference for women: SMR 1.11 (95% CI 0.95, 1.28).

Abbreviations: N, number of included patients; PMR, polymyalgia rheumatica; TAB, temporal artery biopsy; GCA, giant cell arteritis; ACR, American College of Rheumatology; EULAR, European League Against Rheumatism; GC, glucocorticoid; NR, not reported; pts, patients; MRR, mortality rate ratio; RR, Relative risk; SMR, Standard mortality ratio; ASMR, age-standardized mortality rate; CI, confidence interval

Regarding gender differences in survival, we observed a more favorable outcome among male PMR patients, consistent with findings reported by Salvarani et al. [18]. In our study, the survival benefit was most evident in men diagnosed between the ages of 60 and 69. Conversely, women in the same age group exhibited significantly lower survival rates and higher mortality. The reasons for this gender-related disparity remain unclear, and similar findings have not been reported in GCA cohorts [20]. Our study did not investigate causes of death, limiting the ability to explain these differences, which became most pronounced from 15 years after diagnosis and onwards (Fig. 3). The previously reported short-term survival advantage in this cohort, compared to age- and sex-matched controls was not found to be attributed to any specific cause of death until end of follow-up in 1997 [15]. Importantly, the former study did not conduct separate analyses for men and women.

The favorable survival outcomes in PMR-, and possibly GCA-patients are notable, given their association with systemic inflammation, prolonged GC use, a high prevalence of GC-related adverse events, and, in GCA, an increased risk of severe long-term aortic complications [6, 21, 22]. In particular, chronic GC therapy has been linked to increased mortality for other conditions, including RA [23, 24]. One proposed explanation for the paradoxically improved survival in PMR and possibly GCA is enhanced medical surveillance, as regular follow-up may facilitate earlier detection and treatment of comorbidities such as cardiovascular disease [8, 25]. However, similar survival advantages have not been reported in other inflammatory rheumatic conditions [26], suggesting additional contributing factors. Both genetic factors and distinct immunological profiles unique to PMR and GCA patients have also been proposed as potential explanations [27]. A prior study found familial clustering of longevity among PMR and GCA patients, with a significantly higher prevalence of nonagenarian (≥ 90-year-old) mothers and parents overall compared to matched controls [27]. Unfortunately, our study did not have access to parental age data.

The strengths of this study includes the prospective long-term data derived from an inception cohort, which provides unique insights into the natural outcome of PMR over an extended period. As the vast majority of the study population was deceased by the study end, additional follow-up is unlikely to change the results, and our mortality estimates can be regarded as highly reliable. Each patient in the inception cohort was diagnosed by two experienced rheumatologists, were mimicking conditions of PMR were excluded at the time of diagnosis, enhancing the validity of the diagnoses. Finally, the study design likely allowed the inclusion of nearly all incident PMR cases in Aust-Agder County during the inclusion period, as suggested by previous incidence estimates [8, 12].

Our study has several limitations. The 2012 American College of Rheumatology / European League Against Rheumatism criteria for PMR [28] did not exist at the time the PMR patients were included in the cohort from 1987 to 1997, and the applied Bird`s criteria has rather poor specificity for PMR [29, 30]. Previously published data do, however, indicate that our incident PMR cohort had clinical characteristics similar to those described in other large PMR cohorts, both historical and contemporary [31–33]. For example, at time of diagnosis, PMR patients in our study had a mean diagnostic delay of 2.9 months, acute symptom onset in 56%, pain and stiffness in the shoulders in 78%, and mean erythrocyte sedimentation rate and C-reactive protein of 72.0 mm/hr and 68.7 mg/L, respectively [13]. Treatment patterns were also comparable, with a mean initial prednisolone dose of 21.5 mg/day [17]. Unfortunately, gender differences in the baseline parameters were not reported. However, the prevalence of peripheral arthritis in our cohort did not show a statistically significant difference between men and women (53.6% vs. 41.5%, *P* = 0.14) [14]. In a previous publication regarding the PMR cohort, 11 of 202 patients (5.4%) developed RA, and 3 of these were seropositive [14]. Nine of the 11 developed RA more than 3 years after PMR diagnosis. Apart from one patient that had concomitant lung cancer at diagnosis, no other malignancies were diagnosed at presentation and the frequency of malignancy was not found to be increased compared to controls [34]. These findings indicate that misdiagnosis due to malignancy did not represent a marked bias in this study. Due to limited data from medical charts kept on the patient cohort, it was not feasible for us to identify and exclude these cases from our analyses. Finally, the prevalence of large-vessel vasculitis in our PMR cohort may have been underestimated, as these patients were not routinely investigated with imaging studies, which were infrequently part of standard clinical practice when the study was initiated.

In conclusion, to our knowledge this prospective study represents the first to follow a relatively large inception cohort of PMR patients over a long period, with near-complete data on mortality. We found that all-cause mortality for patients with isolated PMR and biopsy-proven GCA was not increased compared to population comparators. Conversely, men diagnosed with PMR had lower all-cause mortality. Our findings align with previous evidence reinforcing that isolated PMR does not significantly impact survival negatively, offering reassurance to both patients and clinicians regarding its long-term prognosis.

## Data Availability

The data underlying this article will be shared on reasonable request to the corresponding author.

## Abbreviations

ACR: American College of Rheumatology
CI: Confidence interval
EULAR: European Alliance of Associations for Rheumatology
GC: Glucocorticoid
GCA: Giant cell arteritis
NPR: National population registry
PMR: Polymyalgia rheumatica
RA: Rheumatoid arthritis
SD: Standard deviation
SMR: Standard mortality ratio
TAB: Temporal artery biopsy
